# A “Common Sense” Surgical Approach to Motor Shoulder Dysfunction Diagnosed as CRPS I/II Following Projectile Injuries to the Regio Deltoidea in the Late Stages of Ballistic Trauma

**DOI:** 10.1055/a-2858-9943

**Published:** 2026-05-04

**Authors:** Alexander A. Gatskiy, Ihor B. Tretyak, Vitaliy I. Tsymbaliuk, Iurii L. Chyrka, Sergii V. Vernygorodskyi

**Affiliations:** 1 Restorative Neurosurgery Department, Romodanov Neurosurgery Institute, Kyiv, Ukraine; 2 National Academy of Sciences of Ukraine, Kyiv, Ukraine; 3 SPINEX Neuro-Orthopedical Clinic, Vinnitsia, Ukraine; 4 Pathological Anatomy Department, Vinnitsia National Pirogov Memorial Medical University, Vinnitsia, Ukraine

**Keywords:** projectile injury, peripheral nerve, axillary nerve, intercostobrachial nerve, CRPS, glenohumeral joint, denervation, nerve transfer

## Abstract

**Background:**

High-velocity projectile injuries to the regio deltoidea (RD) can result in persistent pain and motor shoulder dysfunction (MSD). The underlying mechanisms may involve direct axillary nerve (Ax) trauma or remote nociceptor irritation caused by cavitation effects.

**Objective:**

To characterize the anatomical basis of MSD and to establish a reliable diagnostic and surgical strategy for its management.

**Methods:**

Twenty-seven patients presenting with painful MSD following projectile trauma to the RD were included. Eight patients exhibited deltoid palsy associated with Ax injury, of whom three underwent Ax reconstruction. After positive diagnostic nerve blocks, 25 patients proceeded to denervation of the glenohumeral joint (GHJ): anterior ( *n* = 15) or posterior ( *n* = 3). Surgical procedures included neurotomy of the lateral pectoral nerve ( *n* = 18), suprascapular nerve ( *n* = 3), and Ax ( *n* = 4). In patients with persistent CRPS-like symptoms, neurotomy of the intercostal nerves (ICN) was performed.

**Results:**

Pain relief following Ax surgery was achieved in 25%. Anterior GHJ denervation resulted in 60% pain improvement, whereas posterior showed no significant benefit. RD injuries in 45% cases exhibited CRPS type I-like manifestations. Subsequent ICN neurotomy in 12 patients effectively resolved these symptoms.

**Conclusion:**

High-velocity projectile injuries to the RD may provoke nociceptor irritation at a distance from the entry site through a cavitation mechanism. Direct Ax injury accounted for only 25% of MSD cases. Irritated nociceptors generate CRPS-like symptoms at painful MSD without overt nerve disruption. Targeted neurotomy of the involved nociceptors represents a reasonable therapeutic option for pain alleviation in selected patients.

## Introduction

**Fig. 1:**
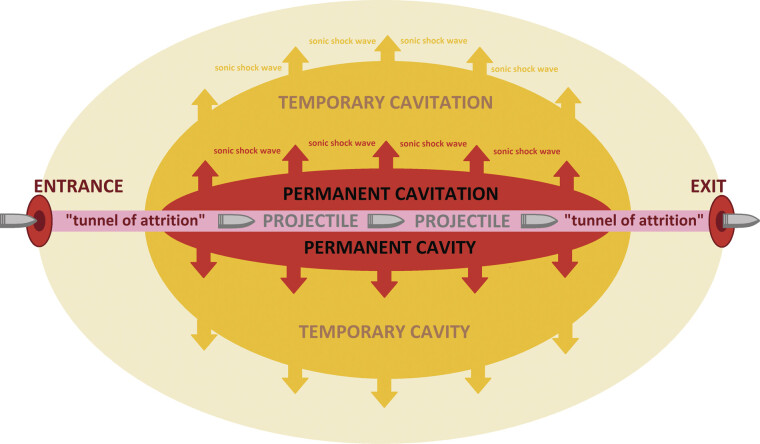
Schematic representation of the mechanisms of high-velocity projectile injury to the tissues in ballistic trauma.

High-velocity projectile injuries in ballistic trauma 1 affecting any anatomical region of the human body produce two major groups of symptoms: 2 (1) in situ: occurring in close proximity to the entrance/exit wounds and along the “tunnel of attrition” 3 ; (2) distant: occurring at a short or relatively long distance from the entrance/exit wounds and the “tunnel of attrition.” 2 In situ symptoms are associated with direct damage to superficial and deep anatomical structures through mechanisms of laceration and crushing, resulting in permanent cavitation 2 ( Fig. 1 ). Distant symptoms result from indirect damage to deep anatomical structures caused by tissue stretching; when tissue elasticity is exceeded, this mechanism also leads to laceration at a distance from the permanent cavity, known as temporary cavitation 2 ( Fig. 1 ).

Regio deltoidea (RD) is a triangular anatomical region outlined by the heads of the deltoid muscle. 4 Compared with the more complex anatomical structure of the shoulder, 5 RD contains a relatively limited variety of structural components. 5 These include the deltoid muscle (the primary mover of the shoulder), tendons (insertion points of the scapulohumeral muscles involved in shoulder motion 5 ), cartilages, 5 and bones, 5 all arranged in a layered manner. 5 RD is devoid of any major motor or sensory nerves and arterial or venous vessels. 5 Under peacetime conditions, the principal clinical manifestation of direct penetrating injury to any layer of RD is loss of active shoulder motion along one axis (and rarely multiple axes), depending on the structure involved. Such motor deficits do not represent a “clinical riddle” and have clear and reliable surgical solutions. 6

In contrast, during wartime, when direct penetrating injuries to RD are mainly caused by high-velocity projectiles, the clinical presentation typically involves a combination of altered motor and sensory (predominantly nociceptive) functions in situ, arising from the anatomical structures within RD, 5 and distant symptoms originating from the neighboring anatomical regions, including the regio axillaris, regio infraclavicularis, and regio supraclavicularis. 5 As a result, these injuries often lack an obvious or reliable surgical solution due to their multifaceted clinical presentation.

Moreover, pain originating from various nonmajor neural structures, whether sensory 7 or sensorimotor, 7 and arising either in situ or distant, frequently becomes the dominant symptom of the injury. In many cases, the absence of clear information regarding the precise pain generator, combined with the lack of involvement of any major neural structures, leads clinicians to interpret this condition as complex regional pain syndrome (CRPS). 8 A CRPS diagnosis, however, makes it exceedingly difficult for a surgeon to identify appropriate targets for primary surgery and for potential secondary, tertiary, or even quaternary procedures. Therefore, these patients require a categorization of symptoms (followed by their grouping) according to the structural origin (anatomy) of the dysfunction, as well as a congruent diagnostic and surgical program (algorithm) aimed at alleviating all existing symptoms with predictable functional outcomes.

The aims of the study were as follows:

1. To define the incidence of primary and secondary motor shoulder dysfunction (MSD) in this series.
2. To identify all neural structures responsible for secondary MSD in this series.
3. To determine neural structures and their prevalence as in situ and distant pain generators in RDa/RDp injury ( Table 1 ).
4. To identify neural structures responsible for CRPS-like symptoms in RDa/RDp injury.
5. To develop an algorithm for the topical diagnosis of secondary MSD.
6. To outline key features of the diagnostic and surgical approach to secondary MSD with CRPS-like manifestations.

**Table 1:** Glossary of “unconventional” a nomenclature used b in this study c

Name	Definition
RDa	Regio deltoidea anterior, a triangular-shaped region with its base inverted cranially, outlined superiorly by the inferior border of the clavicle (basis), medially by the anterior margin of the pars clavicularis of m. deltoideus, and laterally by an imaginary line starting from the anterior edge (tip) of the acromion and extending toward the insertion point of m. deltoideus at the tuberositas deltoidea humeri (a midline dividing the entire regio deltoidea)
RDp	Regio deltoidea posterior, a triangular-shaped region with its base inverted cranially, outlined superiorly by the spina scapulae (basis), medially by the medial margin of the pars scapularis of the m. deltoideus, and laterally by an imaginary line that starts from the posterior edge (tip) of the acromion and extends toward the insertion point of m. deltoideus at the tuberositas deltoidea humeri (a midline dividing the entire regio deltoidea)
Primary motor shoulder dysfunction	Paresis or palsy (at its maximum) of the respective portion of m. deltoideus scored at 2 points or lower on the Medical Research Council Scale (MRC) 9 scale; or a decrease (at its minimum) in range of motion (ROM) 10 during voluntary movement mediated by the respective portion of m. deltoideus along the corresponding axis. Electrophysiology studies (electrodiagnostics (EDX)), according to conventional criteria, 11 confirm dysfunction of any portion of m. deltoideus with respect to the site of injury
Secondary motor shoulder dysfunction	Function of m. deltoideus preserved clinically and according to EDX 11 data; however, passive (with external assistance) or active movement in the glenohumeral joint along any axis (or all axes), with respect to the site of the injury (or irrespective), is impossible due to severe pain (5 points or higher on the Visual Analogue Scale, 12 located in the regio deltoidea anterior, posterior, or both
Superficial pain generators	Nociceptive afferents of different origins (derivatives of sensory and sensorimotor nerves) that terminate within the borders of an injured anatomical region and generate ascending nociceptive (pain) stimuli from superficial layers (skin, superficial fascia) of the respective region
Deep pain generators	Nociceptive afferents of different origins (derivatives of sensory and sensorimotor nerves) that terminate within the borders of an injured anatomical region and generate ascending nociceptive (pain) stimuli from deep layers (ligaments, cartilages, muscles, periosteum, etc.; articular nociceptive afferents)
In situ pain generators	Nociceptive afferents that terminate within superficial or deep structures of an injured anatomical region and provide the main neural supply to its layers
Distant pain generators	Nociceptive afferents that originate in a neighboring anatomical region and, when irritated, generate referred 13 pain sensation in an injured anatomical region; distant nociceptive afferents traverse (intersect) an injured anatomical region and/or provide additional neural supply to its layers

a “Unconventional” indicates that there is currently no supportive literature data regarding the terms, or that the applied terms are not currently included in any standard classification.

b The nomenclature has been adapted to optimally support the decision-making process and the logical flow of the study.

c Despite the use of “unconventional” terminology, all diagnostic and surgical procedures used in this study were fully compliant with the principles of modern evidence-based medicine in the field of peripheral nerve surgery.

## Methods

### Study Type

This was a retrospective, single-center cohort study including 27 consecutive cases treated between 2022 and 2025. Inclusion criteria were:

1. Penetrating projectile injury to the RD.
2. Pain within the borders of the RD (with or without referred pain 13 in neighboring regions) at rest and/or during active or passive movement of the glenohumeral joint (GHJ) along any axis.
3. Absence of soft-tissue defects of the injured upper extremity and absence of acute or chronic infected wounds.
4. No requirement for orthopedic intervention at the time of inclusion, and/or all required orthopedic interventions had been performed previously and were considered successful.
5. Absence of injury-associated pathological fusion (ankylosis) of the GHJ, with passive range of motion (ROM) 10 degrees below the clavicles, not less than 50% compared with the contralateral healthy side.
6. Absence of injury to the infraclavicular brachial plexus and/or arterial or venous vessels.

### Patient Population

Twenty-seven male patients with projectile injuries to the anterior (RDa) and posterior (RDp) deltoid region were included. The mean age was 36.2 years (range: 27–58). Sixteen patients (59%) sustained projectile injuries to the RDa, while eleven patients (41%) sustained injuries to the RDp.

The mean interval from injury to inclusion was 6.2 months (range: 3–14). At the time of inclusion, eight patients (30%) exhibited deltoid muscle palsy, confirmed clinically (according to the Medical Research Council [MRC] Scale 9 ) and by electrodiagnostic (EDX) studies (Local Institutional Protocol comparable to the AANEM Recommended Policy for Electrodiagnostic Medicine 11 ). Two patients had severe paresis (M2 on the MRC scale) of the clavicular part of the deltoid muscle. The remaining six patients showed involvement of the acromial and scapular parts of the deltoid muscle: two demonstrated complete palsy (M0 on the MRC scale), and four showed partial palsy (M1-2 on the MRC scale).

Nineteen patients (73%) were included in the study with no signs of deltoid palsy (M4 on the MRC scale) in either the clavicular part (14 patients) or the acromial and scapular parts (five patients). All patients reported pain within the RD associated with voluntary active or externally assisted passive movement of the GHJ, scored at 5 points or more on the Visual Analogue Scale (VAS 12 ). Fifteen patients (55%) reported resting pain of varying characteristics, including seven patients (44%) after RDa injury and eight patients (73%) after RDp injury.

Thirteen patients (48%) reported pain extending beyond the RD and GHJ projection, involving the infraclavicular region, anterior and lateral thoracic wall, base of the axilla, and posterior and medial surfaces of the arm. Pain originating from these adjacent regions exhibited feature characteristics of both mechanical and tactile allodynia, 14 accompanied by exaggerated sudomotor dysfunction. 15

All patients included in the study had previously been diagnosed with CRPS type I or II (19 and 8 patients, respectively) according to the Budapest Criteria 8 during earlier stages of treatment outside our institution.

Among patients with RDa injury, three had undergone debridement of the anterior surface of the GHJ, and two had received glenoid labrum reconstruction via Bankart or Latarjet procedures. 16 17 Eleven patients with RDp injury had undergone reconstruction of a partially torn rotator cuff using various surgical techniques. 18 None of these orthopedic reconstructions performed prior to referral resulted in pain relief or motor recovery.

Eight patients presenting with deltoid dysfunction (denervation) at the time of inclusion were classified as having primary MSD. In contrast, nineteen patients exhibited shoulder dysfunction exclusively due to pain, with no clinical or EDX evidence of deltoid impairment. 9 11 These cases were categorized as secondary MSD.

### Diagnostic Nerve Blocks

A diagnostic nerve block (DNB) was done under ultrasound guidance and sterile conditions using 2% lidocaine. The volume of the injected anesthetic did not exceed 1.0 mL in order to avoid its spreading into overlapped sensory (i.e., nociceptor) territories. Targets for DNB were determined in a step-wise manner. The most probable (according to Dellon 19 ) articular and/or cutaneous pain generator within the affected surface of the RD was prioritized and blocked first. Anatomical and ultrasound landmarks for each anesthetized nerve were in full accordance with Jankovic et al. 20 Joint pain cessation (50% of initial VAS score) with active or passive shoulder movement, regardless of ROM, was considered as a positive response to DNB. Referred pain 13 cessation (VAS score of 0) in the distribution of the sensory nerve was considered as a positive response to DNB. Surgical ablation followed each positive response to DNB. The interval from positive DNB to surgery did not exceed 14 days.

### Surgical Procedures

Indications for the surgical procedures described below were based on clinical and/or EDX findings and/or intraoperative findings:

1. Clinical and EDX 9 11 evidence of motor dysfunction involving any portion of the deltoid muscle.
2. Technical considerations regarding the suitability of the axillary nerve for any reconstructive procedure based on intraoperative findings.
3. A positive response to diagnostic blocks of superficial and/or deep neural structures adjacent to the corresponding surface of the GHJ, as well as sensory neural trigger points in regions neighboring the corresponding GHJ surfaces.

Independent of their chronological order, all surgical procedures were classified as:

1. Axillary nerve (entire or motor/sensory branches) surgery: highest priority, procedures aimed at eliminating deltoid motor deficit (and associated pain) resulting from direct axillary nerve injury.
2. Joint denervation surgery: second-highest priority, procedures aimed at eliminating GHJ pain by ablation (neurotomy) ^a^ of the articular nociceptive afferents, nerve(s), innervating the affected surface of the GHJ.
3. Cutaneous denervation surgery: additional procedures aimed at eliminating referred pain 13 by ablation (neurotomy) of additional pain generators localized beyond the borders of the injured RD, namely: (1) cutaneous nociceptive afferents of the superficial layers (skin, superficial fascia) of the RD and (2) any wall of the pyramid-shaped axillary fossa.

A patient who underwent a single ablation, ablation combined with reconstruction, or reconstruction targeting a single neural structure exclusively (one nerve: one surgery) was considered to have undergone a single-stage procedure. A multi-stage procedure was defined as a surgery targeting more than one neural structure, regardless of the number of operative stages.

#### Axillary Nerve Surgery

##### Regio Deltoidea Anterior (RDa)

In two patients with injury to the RDa, direct reconstruction of the anterior portion of the axillary nerve (Ax(ant)) was feasible only in one case. During surgical exploration, both the proximal stump and multiple very distal terminal muscular branches were identified and subsequently reconstructed with nerve grafts. In the second case, a substantial muscular and cutaneous tissue defect within the RDa precluded any reconstruction of Ax(ant); the terminal branches had been avulsed together with the soft tissues. Only the release of the proximal stump was possible.

##### Regio Deltoidea Posterior (RDp)

Four patients with injury to the RDp were not candidates for reconstruction of the posterior portion of the axillary nerve (Ax(post)) because it had been avulsed from the surrounding muscular mass. In these cases, release of the proximal stump was combined with neurotomy of the superior lateral cutaneous nerve of the arm and the motor branch to the teres minor muscle. In two additional patients, Ax(post) lacerations were identified; however, the lesions were technically unsuitable for grafting. Therefore, selective Somsak 21 nerve transfer was performed.

#### Joint Denervation Surgery

##### Regio Deltoidea Anterior (RDa)

**Fig. 2:**
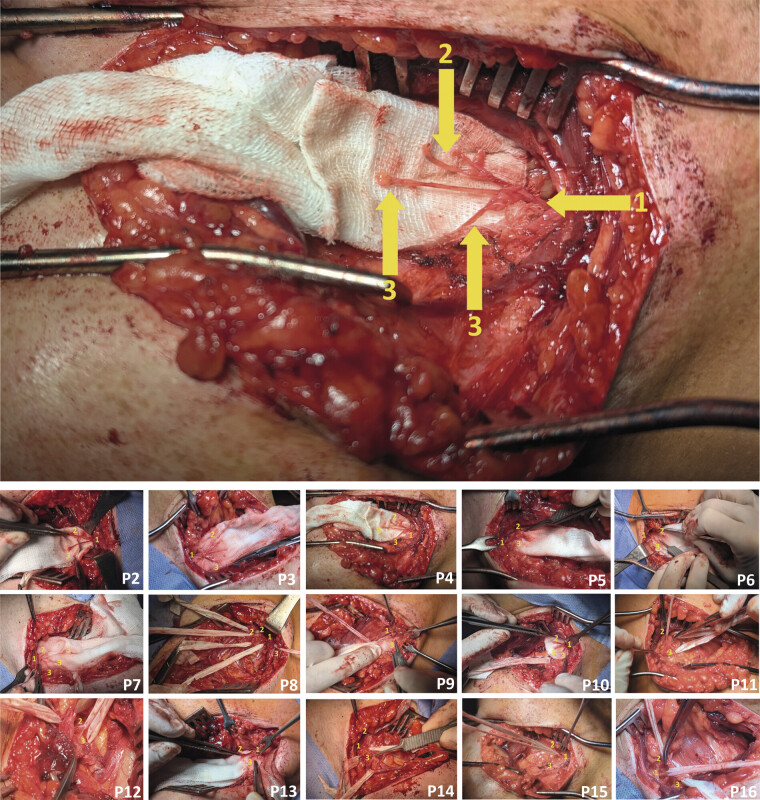
Intraoperative view of neurotomy of the common trunk of n. pectoralis lateralis in the trigonum deltoideopectorale within the fossa axillaris. 1, common trunk of n. pectoralis lateralis; 2, motor branches of n. pectoralis lateralis to m.pectoralis minor; 3, articular branches of n. pectoralis lateralis; P, patient; 2…16, consecutive patient numbers.

Fifteen patients with secondary MSD due to persistent pain localized to the anterior aspect of the shoulder underwent neurotomy of the common trunk of the lateral pectoral nerve (LPN) within the deltopectoral triangle of the axillary fossa ( Fig. 2 ). Four additional patients underwent neurotomy of Ax(ant), followed by immediate Somsak 21 nerve transfer.

##### Regio Deltoidea Posterior (RDp)

Three patients with secondary MSD due to persistent posterior shoulder pain underwent neurotomy of the common trunk of the suprascapular nerve (SS). One case was addressed through an anterior approach in the lateral cervical triangle, followed by direct end-to-end suture. Two cases were treated through a posterior approach in the SS region, followed by Bahm's 21 nerve transfer. Three additional patients underwent neurotomy of the common trunk of the LPN within the deltopectoral triangle of the axillary fossa–partial denervation of the anterior (opposite to an injured RD) surface of the GHJ.

#### Cutaneous Denervation Surgery

##### Regio Deltoidea Anterior (RDa)

**Fig. 3:**
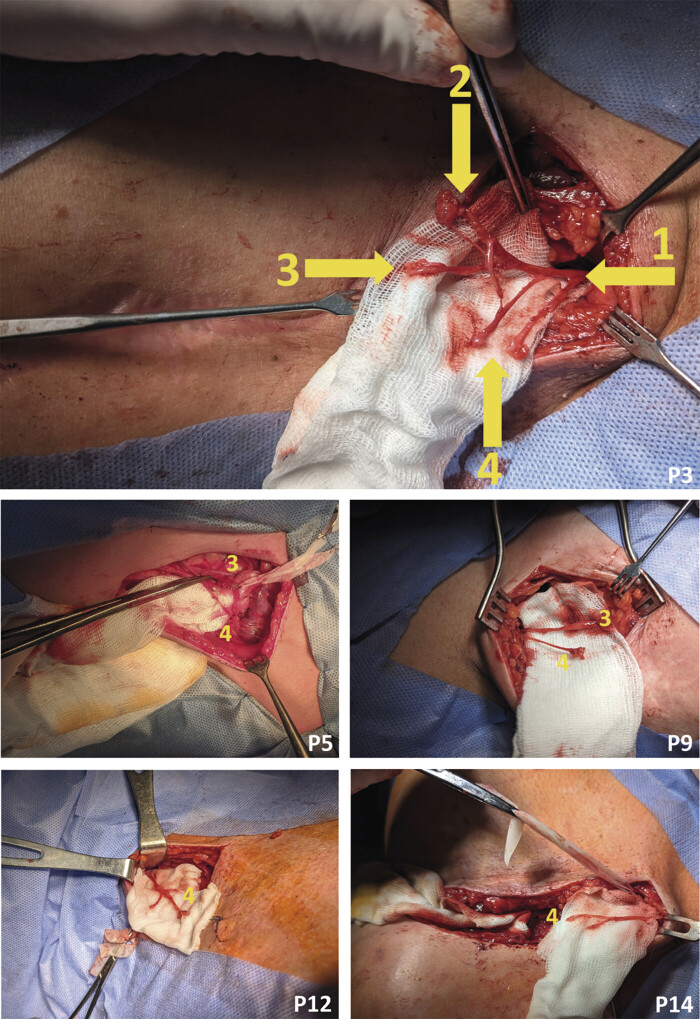
Intraoperative view of neurotomy of the nn. supraclaviculares in the trigonum omoclaviculare within the fossa supraclavicularis. 1, anterior (ventral) ramus of the 4th spinal nerve; 2, rami mediales n. supraclavicularis; 3, rami intermedii n. supraclavicularis; 4, rami laterales n. supraclavicularis; P, patient; 2…1, consecutive patient numbers.

**Fig. 4:**
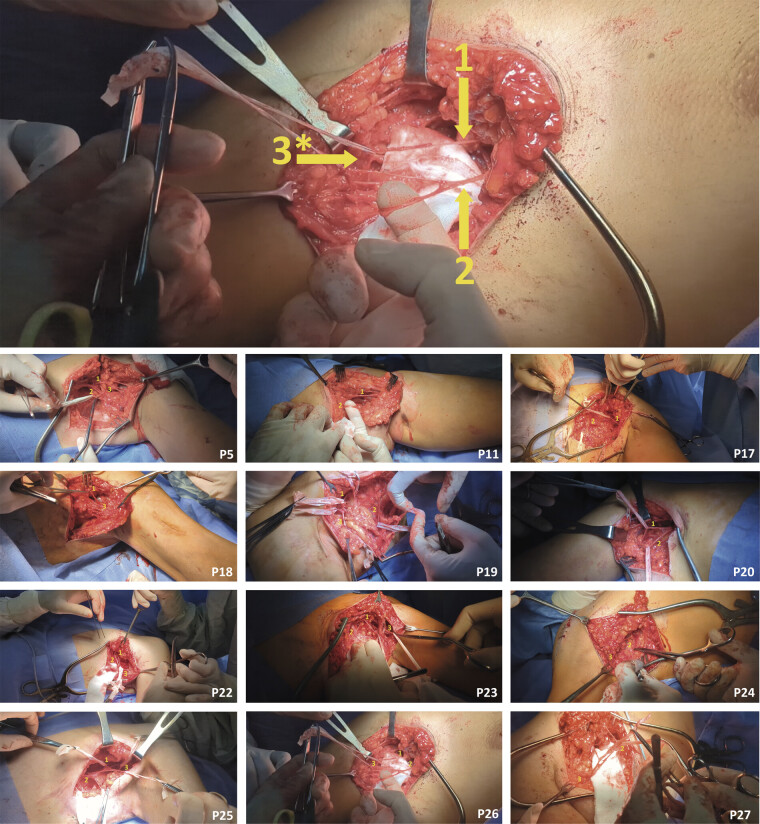
Intraoperative view of neurotomy of rami laterales et mediales nn. intercostales 2–3 and neurectomy of n. intercostobrachialis on the lateral surface of the chest wall and at the base of the fossa axillaris. 1, ramus lateralis n. intercostalis 2; 2, ramus lateralis n. intercostalis 3; 3, n. intercostobrachialis; P, patient; 5…27, a consecutive patient numbers. ^*^ Ramus communicans to n. intercostobrachialis from ramus lateralis n. intercostalis 3.

Five patients underwent neurotomy of the supraclavicular nerves in the omoclavicular triangle of the supraclavicular fossa ( Fig. 3 ). Two patients underwent neurotomy of the lateral and medial branches of the second and third intercostal nerves (ICN) on the lateral surface of the chest wall ( Fig. 4 ).

##### Regio Deltoidea Posterior (RDp)

Ten patients underwent neurotomy of the ICN, as well as neurectomy of the intercostobrachial nerve (ICBN), performed on the lateral surface of the thoracic wall and at the base of the axilla, respectively ( Fig. 4 ).

### Surgical Staging

**Fig. 5:**
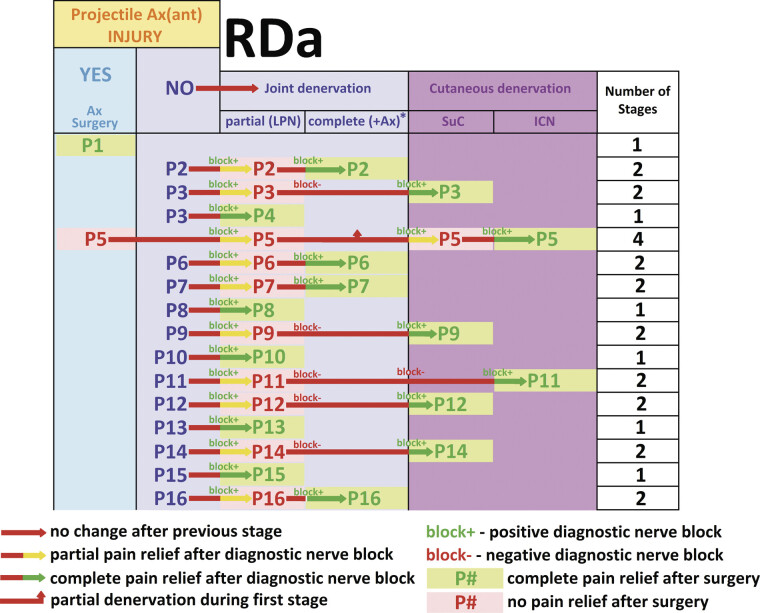
Clinical relevance of diagnostic nerve blocks in the decision-making process preceding each stage of the denervation and the outcomes of surgery in RDa patients. Ax(ant), anterior portion of n. axillaris; GHJ, glenohumeral joint; partial, partial denervation of the anterior surface of GHJ; complete, complete denervation of the anterior surface of GHJ; LPN, ablation of n. pectoralis lateralis; +Ax, surgical ablation (neurotomy) of ramus articularis of the anterior portion of n. axillaris; SuC, surgical ablation (neurotomy) of n. supraclavicularis; ICN, surgical ablation (neurotomy) of nn. intercostales 2–3. ^*^ Surgical ablation (neurotomy) of the articular branch of Ax(ant) exclusively was impossible due to severe scarring/lack of soft tissues in RDa; neurotomy of the entire arterior portion of n. axillaris, followed by immediate reconstruction with Somsak 21 nerve transfer has been performed.

Six patients (37.5%) with RDa injury underwent a single-stage procedure ( Fig. 5 ): one patient underwent axillary nerve surgery, and five patients underwent joint denervation surgery. Ten patients (62.5%) with RDa injury underwent multi-stage treatment ( Fig. 5 ): nine cases required two stages, and one case required three consecutive procedures. Overall, multi-stage procedures comprised five axillary nerve, ten joint denervation, and seven cutaneous denervation surgeries.

**Fig. 6:**
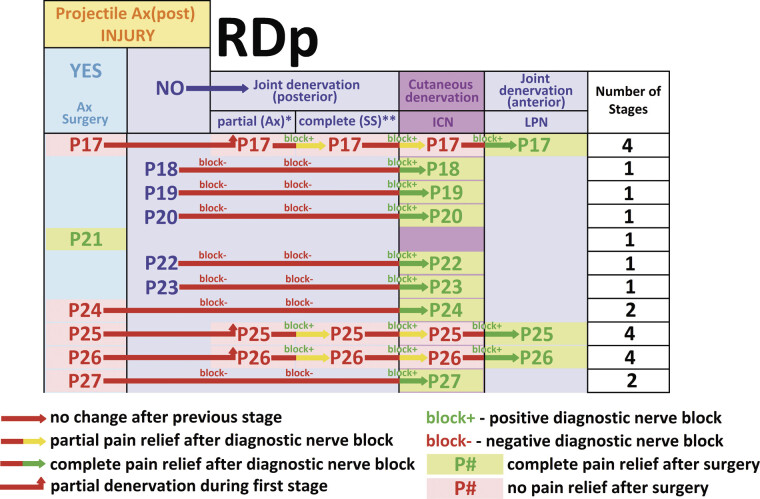
Clinical relevance of diagnostic nerve blocks in the decision-making process preceding each stage of the denervation and the outcomes of surgery in RDp patients. Ax(post), posterior portion of n. axillaris; GHJ, glenohumeral joint; partial, partial denervation of the posterior surface of GHJ; complete, complete denervation of the posterior surface of GHJ; Ax, surgical ablation (neurotomy) of ramus articularis of the posterior portion of n. axillaris; SS, surgical ablation (neurotomy) of n. suprascapularis; SuC, surgical ablation (neurotomy) of n. supraclavicularis; ICN, surgical ablation (neurotomy) of nn. intercostales 2–3. ^*^ Partial denervation of the posterior surface of GHJ with Ax(post) injury. ^**^ Surgical ablation (neurotomy) of the articular branch of SS exclusively was impossible due to severe scarring/lack of soft tissues in RDp; neurotomy of the entire SS followed by immediate reconstruction with Bahm 21 nerve transfer or end-to-end suture has been performed.

Six patients (54.5%) with RDp injury underwent a single-stage procedure ( Fig. 6 ): one patient underwent axillary nerve surgery, and five patients underwent cutaneous denervation surgery. Five patients (45.5%) with RDp injury underwent multi-stage treatment ( Fig. 6 ): two cases were treated in two stages, and three cases required three stages. Overall, multi-stage procedures comprised three axillary nerve, six joint denervation, and three cutaneous denervation surgeries.

The interval from the first stage to each next stage significantly differed due to complicated medical records transferring and patients' logistics (from military to a public health-care system) of any given patient included. The minimal time interval between stages was 1 month, maximal was 6 months.

### Follow-Up Period and Outcome Evaluation

The mean duration of follow-up was 13.2 months (range: 3–26). All patients were examined during follow-up using a standardized protocol. The evaluation included clinical neurological assessment of deltoid muscle strength using the MRC grading system, 9 as well as EDX 11 in patients with injured and reconstructed axillary nerve at 12th months postoperatively and subsequently on demand.

Maximum ROM 10 of the GHJ in the sagittal and frontal planes—corresponding to the function of the relevant portions of the deltoid muscle (shoulder forward flexion, abduction, and extension)—was measured using a manual goniometer once the patient became pain-free (VAS score of 0). Pain intensity was assessed using the VAS. 12 Diagnostic criteria for CRPS I were based on the International Association for the Study of Pain criteria. 22

Patients were considered to experience pain regardless of the VAS score, whereas the absence of pain was defined as a VAS score of 0. Immediate pain relief was defined as a VAS score of 0 at rest following surgery. Late pain relief was defined as a VAS score of 0 at rest and during passive or active shoulder movement, regardless of maximal ROM. 10

Due to the small number of patients, no meaningful statistical analysis was performed. The study design did not include a retrospective control group. Outcome comparison was based on pre- and posttreatment analysis, with patients serving as their own controls.

All procedures involving human participants were conducted in accordance with the ethical standards of the institutional and national research committees and with the 1964 Helsinki Declaration and its later amendments or comparable ethical standards. Informed consent was obtained from all participants included in the study.

## Results

### RDa

Two patients achieved immediate pain relief following LPN-related single-stage surgery ( Table 2 ). In five additional patients, immediate pain relief was achieved after the first stage of LPN ablation, followed by the second and final stage of multi-stage surgery: four cases after SuC ablation and one case after ICN ablation ( Table 2 ). Immediate pain relief in RDa injuries relied exclusively on partial anterior GHJ and cutaneous denervation—ablation of neural structures outside the RDa; Ax(ant) did not contribute to resting pain.

**Table 2:** Incidence of single- and multi-stage ablation of neural pain generators in RDa and RDp

Surgery	RDa					RDp				
	Single-stage	Multi-stage				Single-stage	Multi-stage			
		S1	S2	S3	S4		S1	S2	S3	S4
Axillary nerve surgery						Ax 1	Ax 2			
Joint denervation surgery	LPN 2	LPN 5								
Cutaneous denervation surgery			SuC 4 ICN 1			ICN 5		ICN 2		
Total	7 out of 16 patients					8 out of 11 patients				

Abbreviations: Ax, n. axillaris; ICN, rami laterales et mediales of the corresponding nn. intercostales; LPN, n. pectoralis lateralis; RDa, regio deltoidea anterior; RDp, regio deltoidea posterior; S1–S4, stages of multi-stage surgery; SS, n. suprascapularis; SuC, n. supraclavicularis.

Late pain relief was achieved in six cases following single-stage surgery, corresponding to 37.5% in the RDa subgroup ( Fig. 5 ): in one case after Ax(ant)-related surgery and in five cases after LPN-related surgery. In nine additional patients, late pain relief was achieved after the second and final stage of multi-stage surgery, corresponding to 93.75% efficacy in the RDa subgroup ( Fig. 5 ): four cases after LPN-Ax(ant)-related surgery, four cases after LPN-SuC-related surgery, and one case after LPN-ICN-related surgery. One patient required four consecutive surgeries—Ax(ant), LPN, SuC, and ICN ablation—to achieve late pain relief ( Fig. 5 ). Late pain relief in RDa injuries predominantly relied on a combination of anterior GHJ and cutaneous denervation. Surgery involving injured and noninjured Ax(ant; 1 vs. 4) contributed to relief of the movement-associated pain in 31.3% of cases ( Fig. 5 ).

### RDp

Immediate pain relief was achieved in six patients following single-stage surgery: one case after Ax(post)-related surgery and five cases after ICN-related surgery ( Table 2 ). In two additional patients, immediate relief was achieved after Ax(post)-related surgery, followed by the second and final stage of multi-stage surgery consisting of ICN ablation ( Table 2 ). Ax(post) was identified as a resting pain generator in approximately 12.5% of cases, whereas ICN contributed in 87.5% of cases ( Table 2 ). Immediate pain relief in RDp injuries relied predominantly on cutaneous denervation, reflecting pain generation from neural structures located outside the RDp.

Late pain relief was achieved in six patients following single-stage surgery, corresponding to 54.5% efficacy in the RDp subgroup ( Fig. 6 ): one case after Ax(post)-related surgery and five cases after ICN-related surgery. In two additional patients, late pain relief was achieved after the second and final stage of multi-stage surgery (Ax(post)-ICN-related surgery), corresponding to 72.7% efficacy in the RDp subgroup ( Fig. 6 ). Three patients required four consecutive surgeries—Ax(post), SS, ICN and LPN ablation—to achieve late pain relief ( Fig. 6 ). Late pain relief in RDp injuries predominantly relied on cutaneous denervation of the lateral surface of the thoracic wall and the base of the axilla. No clear contribution of Ax(post) to movement-associated pain had been established ( Fig. 6 ).

Pain relief at rest remained unchanged throughout the follow-up period. Multiaxial shoulder movements were pain-free but did not reach maximal ROM (active or passive) along any given axis. 10 23 In one case of multi-stage surgery, poor recovery of the SS function necessitated a muscular transfer of the ascending part of the trapezius muscle (Elhassan transfer 24 ) to restore external rotation of the shoulder.

The postoperative course following neurotomy of sensory (SuC, ICN) and mixed (Ax, LPN) nerves did not differ from expectations. Neuroma-related pain was elicited in situ upon percussion; however, no persistent neuropathic pain or CRPS-like symptoms 22 were observed. No additional surgical techniques aimed at preventing neuroma formation 25 were utilized in any of the neurotomized nerves.

## Discussion

War-time extremity injuries represent a fundamentally new clinical challenge. Despite substantial progress in limb salvage procedures, 26 effective functional recovery of a severely injured but anatomically preserved extremity remains unlikely. The limited number of publications addressing projectile peripheral nerve injuries (PNI) 27 28 29 30 appears outdated, as the proposed approaches to restoring motor and sensory function no longer reflect current surgical concepts. Most of these studies were conducted long before the zenith of the “nerve transfer era.” 28 Contemporary conceptual approaches 31 to ballistic PNI are based on very small clinical series, 32 33 making it difficult to extrapolate generalized treatment strategies from the reported outcomes.

An effective surgical solution for another major contributor to PNI-associated functional impairment—pain—has yet to be established. Novel strategies, such as targeted muscle reinnervation, 25 regenerative peripheral nerve interfaces, 25 nerve or spinal cord stimulation, 34 as well as nerve reconstruction itself, demonstrate limited practical potential despite promising experimental data. Moreover, in a multi-layered and multi-structural anatomical region, pain may originate from an injured in situ neural structure, an uninjured neural structure at a distance, or a combination of both.

This study provides insight into the management of complex clinical manifestations of primary and secondary (pain-induced) MSD, initially diagnosed as CRPS type I or II, following projectile injuries to the anterior (RDa) and posterior (RDp) regions of the deltoid.

The study findings revealed that among eight patients with confirmed laceration of the anterior or posterior portion of the axillary nerve (Ax), representing primary MSD, only two patients (25%) benefited from Ax-related surgery. In these cases, pain relief at rest and during movement was achieved following nerve grafting and proximal stump release.

**Fig. 7:**
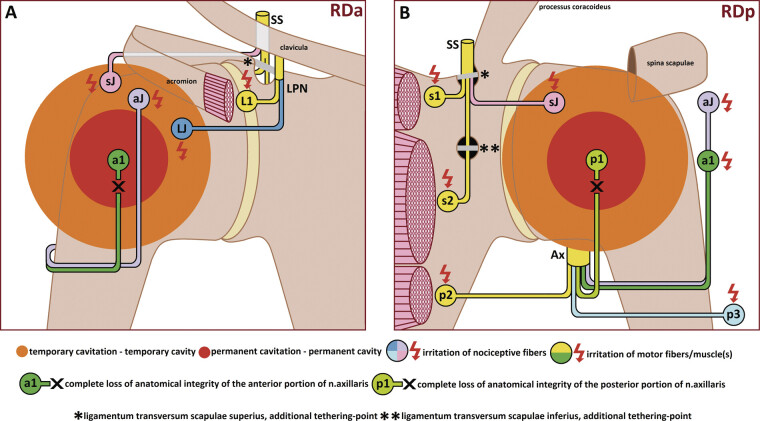
Schematic representation of injury to the neural structures adhered to the surfaces of the GHJ via mechanisms of permanent and temporary cavitation within the borders of RDa and RDp. ( **A** ) RDa(regio deltoidea anterior); ( **B** ) RDp(regio deltoidea posterior); SS, n. suprascapularis; s1, motor branch of n. suprascapularis to m.supraspinatus; s2, motor branch of n. suprascapularis to m.infraspinatus; sJ, articular branch of n. suprascapularis; LPN, n. pectoralis lateralis; LJ, articular branch of n. pectoralis lateralis; Ax, n. axillaris; p1, motor branch of the posterior portion of n. axillaris to pars acromialis et scapularis m.deltoideus; p2, motor branch of the posterior portion of n. axillaris to m.teres minor; p3, n. cutaneus brachii lateralis superior of the posterior portion of n. axillaris; a1, motor branch of the anterior portion of n. axillaris to pars clavicularis m.deltoideus; a2, articular branch of the anterior portion of n. axillaris.

We hypothesize that pain occurring at rest and during movement after projectile-induced complete laceration of either portion of Ax, representing primary MSD, can be rationally explained. High-velocity projectile nerve injury is invariably accompanied by damage to the surrounding soft tissue bed 35 within the borders of the permanent cavity ( Fig. 7A, B —red circle). Secondary injury caused by a sonic shock wave exceeding the elastic limits of soft tissue bed results in the formation of hyperechoic scar tissue surrounding the neural structure, 36 impairing nerve gliding 35 37 or, in severe cases, tethering the nerve stump in an undesirable position. 38

A tethered, injured anterior (ant) or posterior (post) portion of the axillary nerve ( Fig. 7A, B , a1 and p1, respectively), when subjected to compression 39 at rest or excessive stretching 40 during active or passive movement, or both, generates peripheral 40 pain signals. These signals undergo segmental processing 40 and trigger subsequent firing of sensory and motor neurons within reflex circuit pathways at the segmental level 41 of the spinal cord.

The segmental apparatus of the spinal cord may further spread irritation from the injured Ax(ant; Fig. 7A , a1) in a reciprocal manner 42 to nociceptive fibers of the same nerve ( Fig. 7A , aJ), or, conversely, to motor ( Fig. 7B , p1 and p2) and nociceptive ( Fig. 7B , p3) fibers within the uninjured Ax(post), or both simultaneously. Repetitive involuntary muscle activity triggered by uncontrolled neuronal firing further aggravates neural irritation. 42 The cumulative effect of persistent pathological neuronal activity arising from multiple origins forms a “circulus vitiosus,” resulting in a “wandering” pain perception within the cortical regions responsible for the RDa and RDp projections, or both, sometimes without distinct localization. 42

Another six patients in this study with confirmed axillary nerve injury continued to experience pain in the RD even after Ax-related surgery. These patients were retrospectively assigned to the secondary MSD subgroup, which, together with 19 patients without Ax injury, comprised a total of 25 patients.

We assumed that secondary MSD—whether associated with (but not caused by) axillary nerve injury, or occurring independently—shared a very distinct neuroanatomical background, and that the occurrence of pain was governed by the exact same established peripheral, 39 40 segmental, 40 41 and central 42 processes, as well as the hypothesized cumulative mechanism. However, pain was generated by a neural structure located at a distance from the permanent cavity ( Fig. 7A, B —red circle), within the much wider regions affected by the temporary cavitation mechanism 2 ( Fig. 7A, B —orange circle).

The fundamental work by Dellon 19 facilitated identification of these neural structures adherent to the respective surfaces of the GHJ ( Fig. 7A, B ): (1) the articular branch of the SS nerve (sJ), the articular branch of the anterior portion of the axillary nerve (aJ), and the articular branch of the LPN for the anterior surface of the GHJ; (2) sJ for the posterior surface of the GHJ.

Based on DNB targeting GHJ nociceptive innervations, 19 we identified patients who were eligible for ablation (GHJ denervation). Complete pain relief following partial and complete anterior GHJ denervation (10 patients vs. 5 patients) in 15 patients was 50 and 80%, respectively. In contrast, partial and complete posterior GHJ denervation (3 patients vs. 2 patients) resulted in no pain relief. Five patients with RDp injury were not eligible for either partial or complete posterior GHJ denervation due to the negative results of DNBs.

**Fig. 8:**
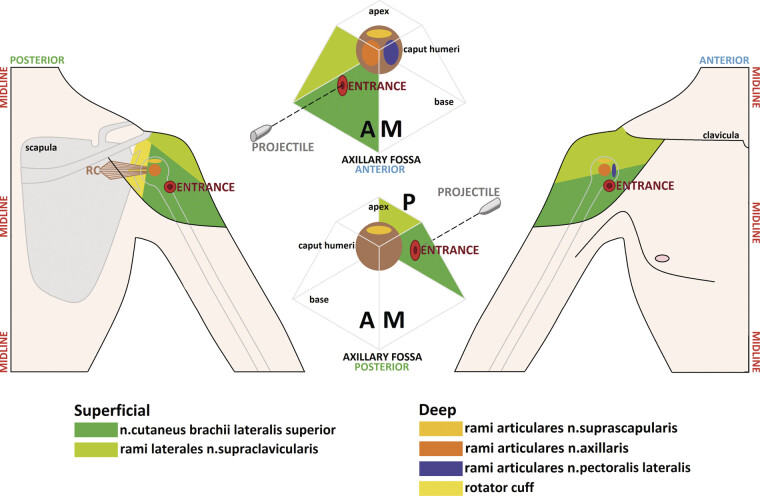
Schematic representation of superficial and deep neural pain generators within the borders of RDa and RDp. apex, roof of the fossa axillaris; base, floor of the fossa axillaris; A, anterior wall of the fossa axillaris; M, medial wall of the fossa axillaris; P, posterior wall of the fossa axillaris.

The poor efficacy of GHJ-targeted interventions was attributed to an initial underestimation of the RDa and RDp as multi-layered anatomical regions. All interventions focused primarily on deep in situ and distant neural structures, whereas pain generators were also located within superficial tissue layers. Consequently, a comprehensive map of all potential superficial and deep neural pain generators within the layered anatomy of the RD was created ( Fig. 8 ).

Superficial pain generators arising from regions neighboring an injured RDa or RDp were identified using ultrasound-guided DNB. One neural structure originating from the cervical plexus in the trigonum omoclaviculare within the supraclavicular fossa and providing sensory innervation to the skin of both RDa and RDp demonstrated a positive response ( Fig. 8 ). This structure was the supraclavicular nerve.

A positive response to SuC DNB was observed exclusively in patients with RDa injury; none of the patients with RDp injury responded positively. Surgical ablation of SuC in five patients with RDa injury resulted in complete pain relief. However, two patients with RDa injury continued to experience severe, persistent pain and did not respond positively to any additional conventional DNB, including those targeting the SS nerve and the superior subscapular nerve. 43

Altogether, clinical data from 12 patients (two with RDa and ten with RDp injury) were interpreted as CRPS type I according to the International Association for the Study of Pain criteria 22 and managed accordingly. Despite unsuccessful attempts to control pain using conventional surgical and later therapeutic approaches, 19 we hypothesized that an unidentified neural background was located in regions neighboring the injured RD.

This hypothesis was supported by the work of Dellon et al. 44 Their retrospective chart review demonstrated that unrecognized nerve compression was the underlying condition in cases of CRPS II misdiagnosed as CRPS I, and that surgical decompression could be an effective remedy. 44

**Fig. 9:**
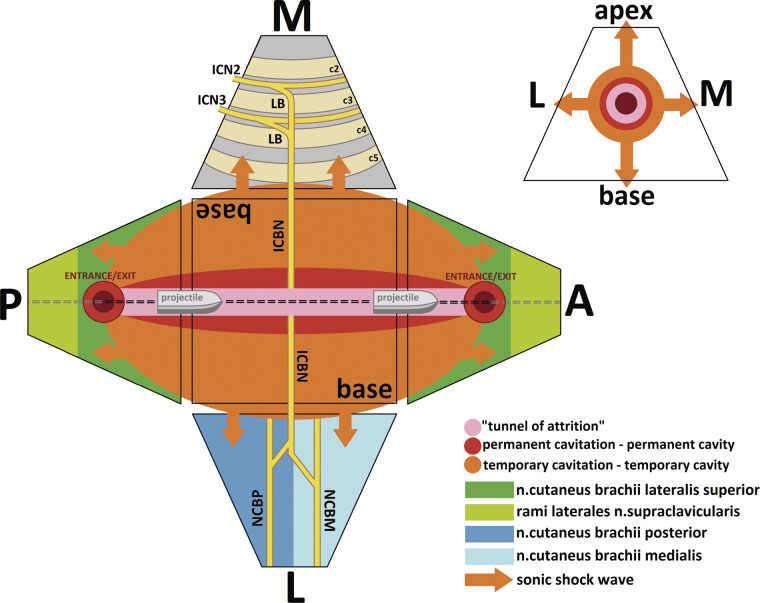
Schematic representation of the pyramid-shaped axillary fossa transformed (“unboxed”) from 3D into 2D plain view. apex, roof of the fossa axillaris; base, floor of the fossa axillaris; A, anterior wall of the fossa axillaris; M. medial wall of the fossa axillaris; L, lateral wall of the fossa axillaris; P, posterior wall of the fossa axillaris; c2…c5, costa 2…5; ICN, n. intercostalis; LB, ramus lateralis of ICN; ICBN, n. intercostobrachialis; NCBP, n. cutaneus brachii posterior; NCBM, n. cutaneus brachii medialis.

At later stages of the study, the main reason for treatment failure became clear. All prior attempts to identify superficial and deep, in situ and distant neural pain generators had been performed exclusively in the anteroposterior plane and along the course of the projectile. In simplified terms, tracing of secondary shock wave injuries to soft tissues, including neural structures, had been conducted in a two-dimensional space. A schematic three-dimensional map of the axillary fossa (another region neighboring the injured RD) allowed tracing of possible directions of the secondary shock wave and identification of potentially involved neural structures ( Fig. 9 ).

Stepwise ultrasound-guided DNB of the second and third ICN at the linea scapularis, and of their lateral and medial branches at the posterior and middle axillary lines, resulted in temporary pain relief at the base of the axilla and along the posterior and medial upper arm. Further analysis of the limited literature related to the ICBN 45 —a direct derivative of the ICN—revealed that the constellation of symptoms observed in the twelve patients diagnosed with CRPS I in this study was similar to those reported in cases of iatrogenic ICBN injury. 45 Moreover, the most prominent CRPS-like clinical manifestation produced by the ICN-ICBN pair in our series—namely, an exaggerated sudomotor reaction—temporarily resolved following distal ICBN block. 46

A combination of convincing facts—the ICN anatomical disposition 45 the ICN-ICBN innervation area, 45 and the positive response to DNB—served as a direct indication for ICN exploration. Twelve patients underwent ICN exploration on the lateral chest wall and at the base of the axilla. Intraoperative findings demonstrated severe scarring, with the ICN adherent to either the posterior wall (RDp injury) or anterior wall (RDa injury) of the axilla. These findings were fully consistent with the peripheral triggering mechanism 39 of pain generation.

ICN neurotomy was then performed, resulting in complete pain relief in all patients, with full resolution of CRPSI-like symptoms. Thus, the painful condition observed in these twelve patients with RDa/RDp injuries represented previously undiagnosed cases of CRPS II, thereby confirming the hypothesis proposed by Dellon et al 44 during the course of this study.

In summary, the diagnosis of the multifaceted late-stage manifestations of projectile injuries to the RD in ballistic trauma should focus on the precise identification of the neural structure(s) responsible for motor dysfunction, pain-related symptoms, or mixed etiology. Given the highly specific mechanism of injury—projectile trauma—the true neural origin of dysfunction is often difficult to localize using conventional clinical neurological examination and EDX studies. Since pain predominated in the clinical picture, a stepwise identification of all neural pain afferents through ultrasound-guided DNB within injured and uninjured neighboring regions remained the only relevant diagnostic and short-term therapeutic approach.

Based on the outcomes of this study, once the neural source(s) of pain had been identified, the surgical solution employed, despite being effective in achieving pain relief, strongly contradicted the generally accepted principle 38 of peripheral nerve surgery—namely, reconstruction. Instead, this “common sense” surgical approach favored destruction of the nociceptive peripheral pathways through neurotomy (ablation) of sensory or mixed nerves. In the latter case, immediate reconstruction of the motor component was required to prevent additional iatrogenic motor deficits in the long term.

Limitations of the study include: (1) the small number of patients; (2) variability in axillary nerve injury; (3) variability in the clinical neurological presentation among included patients; (4) inclusion of patients with and without CRPS-like manifestations; (5) the lack of a generalized and standardized diagnostic and surgical approach for RD-injuries during the early stages of the study.

## Conclusion

In our series, the incidence of primary and secondary MSD was 7.5 and 92.5%, respectively. Neural structures responsible for secondary MSD included the uninjured anterior portion of the axillary nerve (n. axillaris), the LPN (n. pectoralis lateralis), the SS nerve (n. suprascapularis), the suprascapular nerves (nn. supraclaviculares), and the medial and lateral branches of the second and third ICN (rami mediales et laterales of nn. intercostales 2–3).

For RDa injuries, the most probable in situ deep pain generator is the LPN, while the most likely distant superficial pain generators are the supraclavicular nerves. In RDp injuries, the most probable distant superficial pain generators are the lateral branches of the second and third ICN. ICNs play a key role in shaping CRPS-like symptoms in both RDa and RDp injuries.

DNB should always precede the selection of any neural structure for ablation and should follow a stepwise approach: from superficial to deep structures and from in situ to distant structures. As CRPS-like manifestations are predominantly produced by distant neural structures, diagnostic blocks followed by ablation should precede any surgical procedures involving neural structures within the injured RD.
